# The Treasure Vault Can be Opened: Large-Scale Genome Skimming Works Well Using Herbarium and Silica Gel Dried Material

**DOI:** 10.3390/plants9040432

**Published:** 2020-04-01

**Authors:** Inger Greve Alsos, Sebastien Lavergne, Marie Kristine Føreid Merkel, Marti Boleda, Youri Lammers, Adriana Alberti, Charles Pouchon, France Denoeud, Iva Pitelkova, Mihai Pușcaș, Cristina Roquet, Bogdan-Iuliu Hurdu, Wilfried Thuiller, Niklaus E. Zimmermann, Peter M. Hollingsworth, Eric Coissac

**Affiliations:** 1 Tromsø Museum, UiT—The Arctic University of Norway, N-9037 Tromsø, Norway; marie.f.merkel@uit.no (M.K.F.M.); youri.lammers@uit.no (Y.L.); iva.h.pitelkova@uit.no (I.P.); 2 LECA, Univ. Grenoble Alpes, Univ. Savoie Mont Blanc, CNRS, F-38000 Grenoble, France; sebastien.lavergne@univ-grenoble-alpes.fr (S.L.); mboleda1@xtec.cat (M.B.); charles.pouchon@univ-grenoble-alpes.fr (C.P.); cristina.roquet@uab.cat (C.R.); wilfried.thuiller@univ-grenoble-alpes.fr (W.T.); 3 Génomique Métabolique, Genoscope, Institut François Jacob, CEA, CNRS, Univ Evry, Université Paris-Saclay, 91057 Evry, France; aalberti@genoscope.cns.fr (A.A.); fdenoeud@genoscope.cns.fr (F.D.); 4 A. Borza Botanical Garden and Faculty of Biology and Geology, Babeș-Bolyai University, 400015 Cluj-Napoca, Romania; mihai.puscas@ubbcluj.ro; 5 Systematics and Evolution of Vascular Plants (UAB)—Associated Unit to CSIC, Departament de Biologia Animal, Biologia Vegetal i Ecologia, Facultat de Biociències, Universitat Autònoma de Barcelona, ES-08193 Bellaterra, Spain; 6 Institute of Biological Research, National Institute of Research and Development for Biological Sciences, 48 Republicii Street, 400015 Cluj-Napoca, Romania; bogdan.hurdu@icbcluj.ro; 7 Swiss Federal Research Institute WSL, 8903 Birmensdorf, Switzerland; niklaus.zimmermann@wsl.ch; 8 Royal Botanic Garden Edinburgh, Edinburgh EH3 5LR, UK; PHollingsworth@rbge.org.uk

**Keywords:** alpine, chloroplast DNA, environmental DNA, ITS, *matK*, nuclear ribosomal DNA, plant DNA barcode, phylogenomic, polar, *rbcL*

## Abstract

Genome skimming has the potential for generating large data sets for DNA barcoding and wider biodiversity genomic studies, particularly via the assembly and annotation of full chloroplast (cpDNA) and nuclear ribosomal DNA (nrDNA) sequences. We compare the success of genome skims of 2051 herbarium specimens from Norway/Polar regions with 4604 freshly collected, silica gel dried specimens mainly from the European Alps and the Carpathians. Overall, we were able to assemble the full chloroplast genome for 67% of the samples and the full nrDNA cluster for 86%. Average insert length, cover and full cpDNA and rDNA assembly were considerably higher for silica gel dried than herbarium-preserved material. However, complete plastid genomes were still assembled for 54% of herbarium samples compared to 70% of silica dried samples. Moreover, there was comparable recovery of coding genes from both tissue sources (121 for silica gel dried and 118 for herbarium material) and only minor differences in assembly success of standard barcodes between silica dried (89% ITS2, 96% *matK* and *rbcL*) and herbarium material (87% ITS2, 98% *matK* and *rbcL*). The success rate was > 90% for all three markers in 1034 of 1036 genera in 160 families, and only Boraginaceae worked poorly, with 7 genera failing. Our study shows that large-scale genome skims are feasible and work well across most of the land plant families and genera we tested, independently of material type. It is therefore an efficient method for increasing the availability of plant biodiversity genomic data to support a multitude of downstream applications.

## 1. Introduction

Genetic and genomic data are of critical importance for many applications, including species delimitation [1,2,3], studies on evolution and phylogenies [4,5,6], biodiversity assessments and conservation [7,8], reconstructions of past plant communities [9,10,11], or for more applied tasks such as forensics [12,13], pollination and food web studies [14,15,16] and monitoring of invasive species [17]. While many of these tasks can be undertaken by sequencing plastid or rDNA amplicons [1,2,18,19], increasing emphasis has been given to the potential of using genomic data for DNA barcoding and wider phylogenomic studies [4,20,21,22,23,24]. One key approach for gathering large scale genomic data from plants is genome skimming which consists of shallow pass shotgun sequencing [23,25,26,27]. The major advantage of genome skimming is the large amount of genetic information it provides. Genome skims notably allow for simultaneous assembly of both nuclear ribosomal and plastid DNA. Thus, a single analysis may provide complete plastid and ribosomal assemblies including all the plastid and nuclear ribosomal markers that have been used in plant DNA barcoding (e.g., the plastid genes *matK* and *rbcL* [1], plastid spacers/introns such as *trnH-psbA* and *trnL*, as well as the nuclear ribosomal regions ITS1 and ITS2, see also further discussion of plant barcodes [2,18,20]. 

The use of the complete chloroplast genome as a standard barcode has been repeatedly suggested [23,28,29,30] because of its capacity to increase the resolution at lower taxonomic levels in plants [31]. It is also a useful information source for deeper level phylogenetic studies [4]. Most chloroplast genomes are 110–160 kbp, a size that, on the one hand, provides much more information than a few loci and, on the other hand, allows the chloroplast genome to more easily be sequenced and assembled than the much larger nuclear genome. Moreover, when genome skimming approaches are used, the problem of nonuniversal primer sites that have been a limitation for several of the most use markers as *matK*, ITS1 and ITS2 [1,2,20], is avoided. However, the structure and complexity of the chloroplast varies [32], and especially taxa with chloroplast genomes harbouring many repeats are, according to our experience, challenging to assemble and therefore to annotate. Also, in some genera, species may not be distinguishable by chloroplasts due to recent alloploid origins, chloroplast sharing, or hybrid speciation [2]. Nuclear ribosomal DNA is a good complement to the chloroplast genome, as it includes the frequently used and rapidly evolving markers ITS1 and ITS2, as well as the more conserved 18S, 5.8S and 28S [33,34]. 

Generating large scale data-sets involving thousands of samples is a major effort, even with standard amplicon sequencing (e.g., building DNA barcoding reference libraries for regional floras [35,36]). There is thus considerable interest in developing approaches to increase the number of loci recovered from plant samples using a method that is scalable over multiple individuals of multiple species, while remaining tractable and manageable at a scale of many thousands of samples. Two recent studies that have tackled this using genome skimming and have generated large-scale genomic data from plants, showing the potential to extend sampling coverage to the scale of regional floras, the first from China (*n* = 1659) [4], the second from Australia (*n* = 672) [37]. Further studies are required to refine protocols and assess which approaches result in efficient and cost-effective recovery of data. Of particular importance, is the development and testing of informatics pipelines across diverse sample sets, and developing robust laboratory protocols that cope with the inevitable heterogeneity of tissue type and quality that is found in large scale studies. 

A very important, but potentially challenging source of tissue for large scale studies are the plant collections housed in the world’s herbaria. They contain all described species of multicellular plants worldwide including their type specimens, as well as both species that are extinct or not yet described [38,39]. They represent several hundred years of global efforts in collecting, describing and identifying plants in both easily accessible and more remote areas [26,40]. Using herbarium collections for large scale genome skimming thus offers the opportunity to open the ‘treasure vault’ that these specimens represent [41,42]. However, the quality and quantity of DNA found in herbarium specimens depends on conditions during collection and storage, which is, in general, lower than for freshly collected plant material followed by immediate drying in silica gel or freezing [43]. Low quantity and quality of DNA from the outset can affect all downward steps such as sequencing success, assembly and annotation, and may therefore affect the overall success of a large scale project. However, genome skimming methods have improved and several recent studies have shown that it is possible to extract sufficient quality and quantity of herbarium material for retrieval of partial or full complete plastid genomes [41,44,45,46].

In this study we focus on the practicalities of large-scale genome skimming. We share experience gained from two large-scale projects involving several thousands of species to guide future deployment of genome skim sequencing to understand plant biodiversity. The first project (PhyloAlps including PhyloCarpates) is focused on the European Alps and the Carpathians, and is mainly based on freshly collected leaf material dried in silica gel. The second (PhyloNorway), is mainly based on herbarium material from Norway and the Arctic region. Except for a modification in the extraction protocol for the herbarium material, these two projects use the same methods and therefore also allow for herbarium material to be evaluated as a cost-efficient source for large scale genome skimming. Specifically, we evaluate (1) the quality of the DNA recovered, and the success of genome skimming of herbarium and of silica gel dried material, (2) the recovery of standard plant barcodes from the genome skim data, and (3) the effect of sample age and time of growing season on assembling the full chloroplast from herbarium material. 

## 2. Results

### 2.1. The Total Dataset

**Figure 1:**
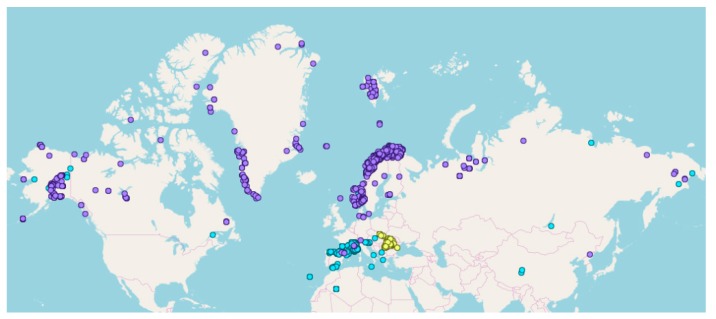
Collection sites for the projects PhyloAlps (blue), PhyloCarpates (yellow) and PhyloNorway (purple).

Overall, 6655 specimens of 5575 taxa (species, subspecies and a few hybrids) belonging to 161 families were sequenced (Table 1). These consisted of 141 families of angiosperms (6469 specimens, 5444 taxa, 997 genera), 4 families of gymnosperms (41 specimens, 31 taxa, 10 genera), and 16 families of ferns (Polypodiopsida: 145 specimens, 100 taxa, 30 genera) (Supplementary Appendix 1). Of these, 4604 were based on fresh leaf tissue collected for the PhyloAlps (4280) and the PhyloCarpates (324) projects and, 2051 were taken from herbarium material sampled for the PhyloNorway project (Table 1). The majority of the specimens were collected in the Alps and Norway, although other arctic and alpine areas were also sampled (Figure 1, Supplementary Appendix 1).

**Figure 2:**
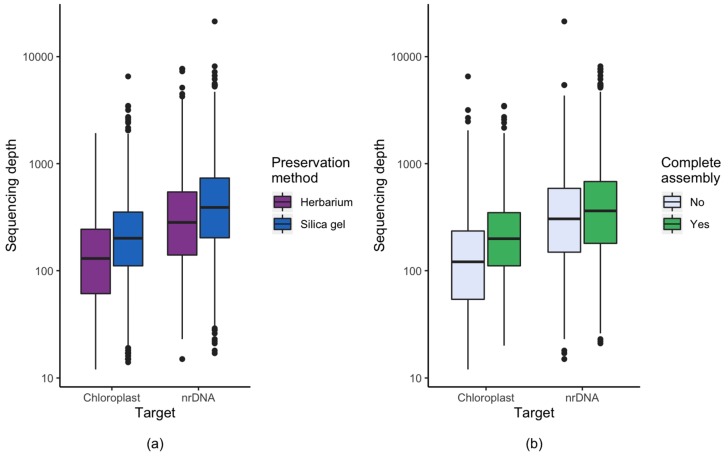
Chloroplast and nuclear ribosomal sequencing depth (the average number of reads representing a given nucleotide) of the total dataset of 4604 freshly collected and silica gel dried material from the Alps and the Carpathians (“Silica gel”) and 2051 herbarium specimens from Norway and polar regions (“Herbarium”). (**a**) Effect of preservation methods on sequencing depth. (**b**) Sequencing depth in relation to complete assembly success for herbarium and silica gel dried material combined. Note that the y-axis is on a logarithmic scale.

**Figure 3:**
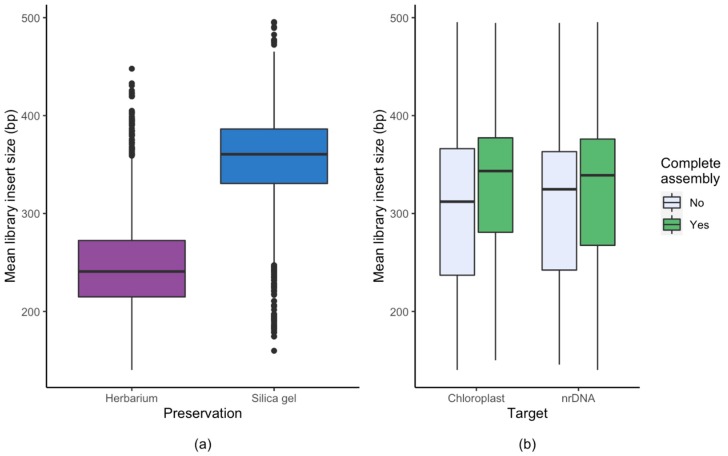
Library insert size (the length of the DNA fragment sequenced) for (**a**) herbarium and silica gel dried material and (**b**) success of complete assembly of chloroplast and nrDNA for herbarium and silica gel dried material combined.

For the chloroplast genome, the average sequencing depth (the average number of reads representing a given nucleotide; also referred to as read depth or average cover) of silica gel dried material was 1.7 times higher than that of herbarium material, and this difference was highly significant (Mann-Whitney *p* = 1.8 × 10^−88^). Similarly, the average sequencing depth of silica gel dried material was 1.5 times higher than that of herbarium material for nrDNA cluster (Mann-Whitney *p* = 3.4 × 10^−5^) (Figure 2). Also, the average library insert size (the length of the DNA fragment sequenced) was on average 100 bp longer for silica dried material compared to herbarium material (Figure 3). The effect of insert size on assembly success was significant in both the chloroplast (Mann-Whitney *p* = 1.7 × 10^−31^) and the nrDNA cluster (Mann-Whitney *p* = 5.4 × 10^−7^, Table 1, Figure 3).

**Figure 4:**
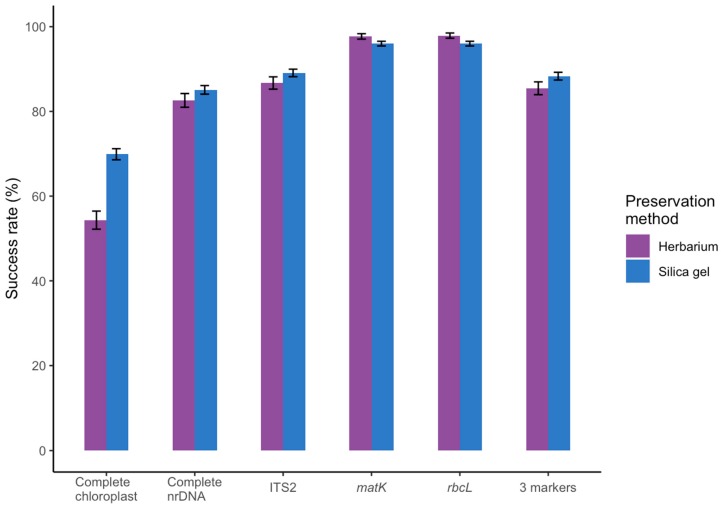
Sequencing success of the complete chloroplast and nrDNA clusters, the standard chloroplast barcodes *rbcL* and *matK*, the optional nuclear ribosomal barcode ITS2, and all three barcodes for freshly collected silica dried (*n* = 4604) and herbarium material (*n* = 2051).

The success rate of assembling the full chloroplast was considerably higher for silica gel dried 70%) than for herbarium material (54%) (Mann-Whitney *p* = 1.73 × 10^−13^, Figure 4). This had, however, little effect on the number of genes assembled as we were able to assemble 121 and 118 chloroplast genes for silica gel dried and herbarium material respectively, using the global assembler. This included on average 77 and 77 Coding Sequences (CDS), 6.6 and 7.1 rDNA and 35.1 and 36.3 tRNA genes for silica gel dried and for herbarium material, respectively. The success rate of assembling the full nrDNA cluster was only slightly higher for silica gel dried (85%) than for herbarium material (83%) (Mann-Whitney *p* = 3.36 × 10^−7^, Figure 4).

For the global assembly method, the success rate for recovering *matK* was higher for silica gel dried (77%) than for herbarium (68%) materials (Fisher *p* = 3.0 × 10^−14^). This rate increased for both materials when we applied the targeted marker assembly leading to similar success rates of 96% and 98%, respectively, for silica gel dried and herbarium material (Fisher *p* = 3.7 × 10^−2^). Similarly, the initial success rate for *rbcL* was higher for silica gel dried (78%) than for herbarium (70%) material (Fisher *p* = 1.5 × 10^−11^), rising to 96% and 98%, respectively, when using the targeted assembler (Fisher *p* = 1.05 × 10^−2^) (Figure 4). For ITS2, only the global assembly was used and the success rate was marginally higher for silica gel dried (89%) than for herbarium (87%) material (Fisher *p* = 5.4 × 10^−3^). All three markers were successfully obtained in 86% for herbarium and 88% for silica gel dried material, and at least one marker was successfully sequenced in all families (*n* = 161) and all genera (*n* = 1047) except for one genus of Alismataceae (*Luronium*, *n* = 1), 7 genera of Boraginaceae (see below), and one of Onagraceae (*Clarkia*, *n* = 1. Supplementary Appendix 1). 

### 2.2. Results For Recovery of Standard Barcode Loci From Large Families

There were 43 families that contained a minimum of 20 taxa each across the projects, thus contributing the majority of specimens across the total dataset (*n* = 5893 samples: 4057 based on silica gel and 1836 on herbarium material). Of these, all families had 10 or more taxa in the PhyloAlps dataset and 32 families had 10 or more taxa in the PhyloNorway dataset. The following families had less than 10 taxa in PhyloNorway: Amarylliaceae (7), Cistaceae (3), Crassulaceae (9), Euphorbiaceae (9), Hyacintaceae (6), Hypericaceae (6), Iridaceae (3), Liliaceae (6), Linaceae (2), Scrophulariaceae (2) and Solanaceae (8): which all had 100% success for *rbcL* and *matK* after targeted assembly, except for Amarylliaceae (6 out of 7 specimens for both marker) and Crassualaceae (11 out of 12 specimens for both markers). 

**Figure 5:**
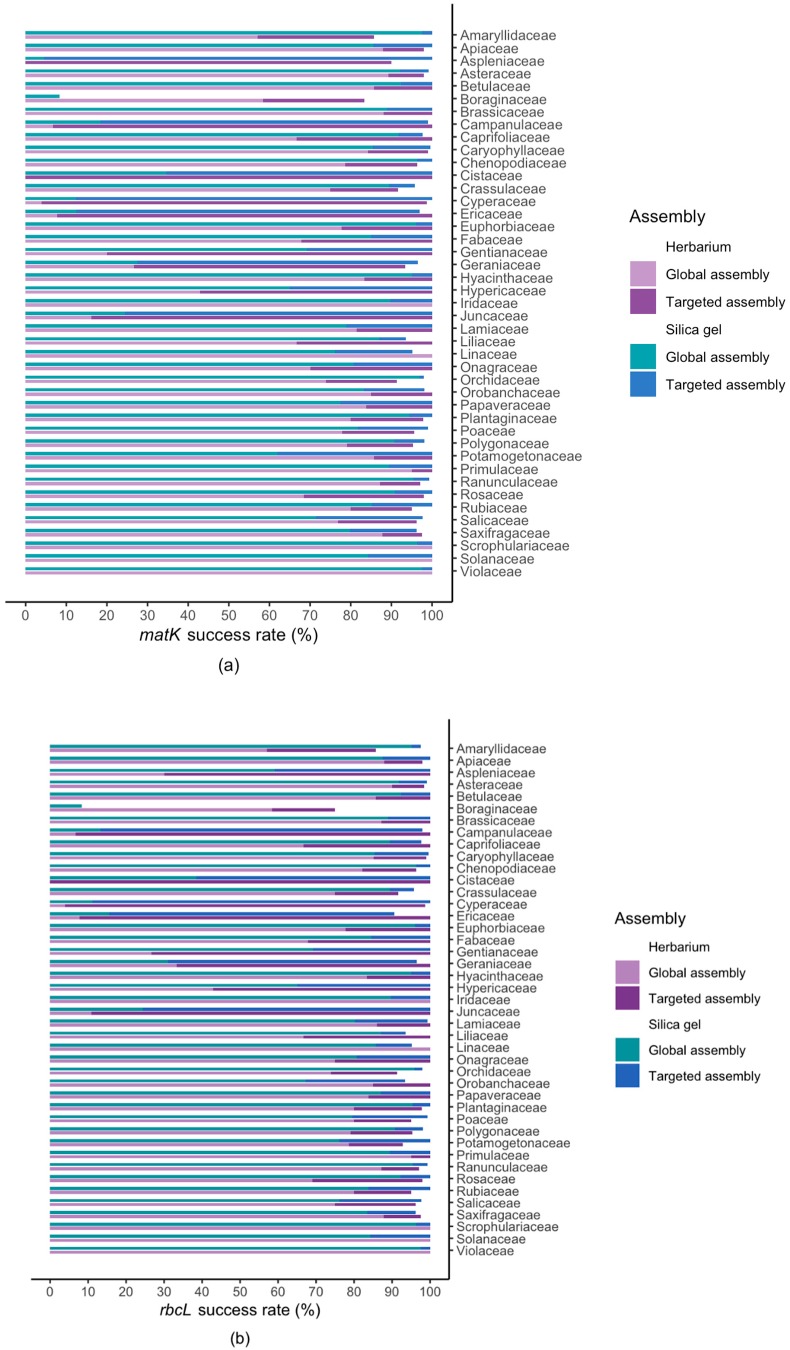

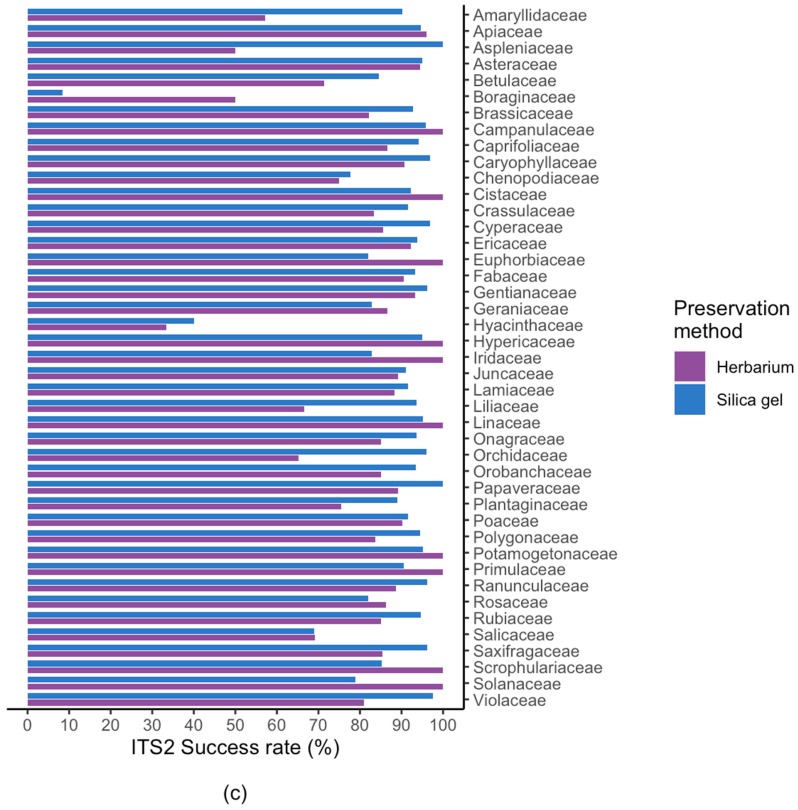
Sequencing success for 43 families with a minimum of 20 taxa available across the studied specimens based on freshly collected silica gel dried (Alps and Carpathians) and herbarium (Norway) material. (**a**) *matK*, (**b**) *rbcL* and (**c**) ITS2.

For the global assembly of the standard barcodes *rbcL*, *matK* and ITS2, the success rate for large families was higher for silica gel dried material than for herbarium material (dark colours in Figure 5). This in part reflects the issue that the assembly of the full chloroplast was problematic in some species-rich families such as Campanulaceae, Cyperaceae and Ericaceae. This was mainly due to the global assembler having difficulties dealing with the highly repeated structure of the chloroplast in these families. However, following the targeted assembly of *rbcL* and *matK*, these differences almost disappeared, and all families except for Amarylliaceae and Boraginaceae had higher than 90% success rate for both herbarium and silica gel dried material. There were close similarities in success rate between *rbcL* and *matK*, whereas the success rate of ITS2 sometimes showed deviating patterns (Figure 5).

For Boraginaceae, the success rate was reasonably high for herbarium material (83% for *matK* and 75% for *rbcL*, *n* = 12) whereas it was only (8% for both markers) for silica gel dried material (*n* = 156). Similarly, the ITS2 success rate was higher for herbarium (50%) than for silica gel dried material (8%). It is important to note that there were partly different genera represented for Boraginaceae in the silica and herbarium samples. The assembly failed for the following Boraginaceae genera: *Alkanna* (*n* = 2), *Asperugo* (*n* = 2), *Borago* (*n* = 2), *Eritrichium* (*n* = 4), *Lappula* (*n* = 4), *Lithodora* (*n* = 2), *Neatostema* (*n* = 2). Among these, only *Eritrichium* was represented in the herbarium material (Supplementary Appendix 1). For some genera that were represented in both herbarium and silica gel dried material, the relative success rate was higher for herbarium than silica, e.g., *Cynoglossum* (1 of 2 herbarium, 0 of 11 silica gel) and *Myosotis* (3 of 3 herbarium, 0 of 31 silica gel).

### 2.3. Effect of Sample Age and Time of Season

**Figure 6:**
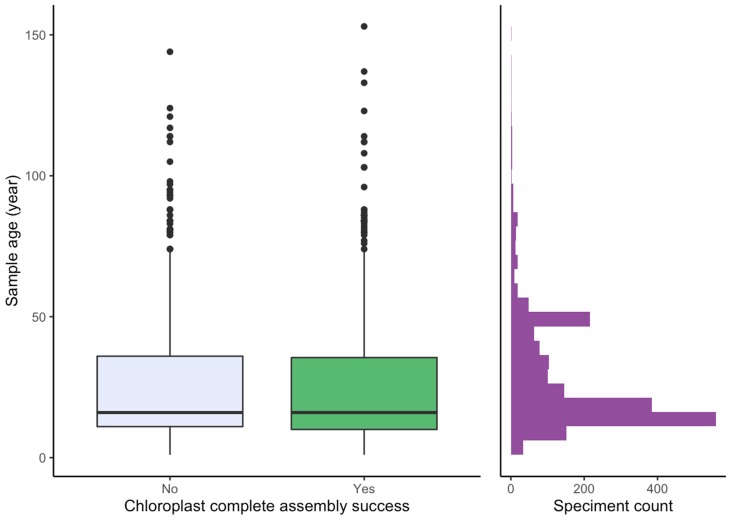
Sequencing success for herbarium material in relation to age.

We had a documented exact age for 2022 herbarium specimens. The majority were less than 20 years old (1166 samples) and only 165 samples were older than 50 years. The maximum age was 153 years old and the full chloroplast of this specimen was assembled. There was no effect of age on chloroplast assembly success (Mann-Whitney *p* = 0.259, Figure 6). Similarly, there was no effect of time of season on chloroplast assembly success (Mann-Whitney *p* = 0.367).

## 3. Discussion 

The overall high success rate clearly shows that large-scale genome skims are now feasible for plant biodiversity genomic studies. This work was undertaken in relatively small laboratories with 1–2 technicians, indicating that large scale genome skimming is feasible even for small laboratories. This clearly opens many future research directions and capacities for many laboratories. 

### 3.1. Effect of Starting Material

While the success rate for assembling the full chloroplast and nrDNA cluster was clearly higher in silica gel dried than herbarium material, this mainly affected the global assembly and had little effect on the ability to retrieve large suites of coding genes. Thus, if retrieving targeted loci is the main goal, herbarium material has about as equal a success rate as does silica gel dried material. This shows that the treasure vault may be opened [40,41], which is a great advantage compared to collecting fresh material for regional barcode projects, because field campaigns are labour- and cost-intensive and require subsequent expert identification [47].

For studies on chloroplast structure, the increased coverage and insert length seen in silica gel dried material likely increased the assembly success. The insert length of silica gel dried material was limited by the size of DNA fragments by sonication (350 bp), whereas the insert length for herbarium material was shorter (250) probably due to degradation during drying and/or storage. There is potential for increasing the success of herbarium material by using an improved extraction protocol [48] or library preparation protocol [49,50], but this is not likely to improve the insert length. For nrDNA clusters, where the average cover was higher due to the higher number of copies in the cell, the assembly success was also higher. This indicates that the sequencing depth was more important than the insert length, and increasing the sequencing effort of the problematic sequencing libraries (we usually target 6 million of reads per library) will automatically increase the sequencing depth of chloroplast, and will most likely resolve the problem related to some herbarium specimens. A sequencing depth of about 90x or higher is sufficient for most families (Figure 2), whereas families with complex chloroplast structure, such as Ericaceae, Cyperaceae and Campanulaceae, may need either deeper sequencing or may require alternative assembly methods.

### 3.2. Success Rate Compared to Amplicon-based DNA Barcoding 

Our success rate for chloroplast loci (96–98%) and ITS2 (87–89%) is similar to what has been recorded for 672 herbarium samples from Australia [37]. In contrast, success rate based on large scale amplicon-based sequencing is generally lower. For example, the success rate for the first regional flora (Wales) was 57% for *matK* (*n* = 2419 specimens) and 77% for *rbcL* (*n* = 3304) [36]. Similarly, the success rate for 3176 specimens of herbarium material from Finland was 79% and 55% for *rbcL* and *matK*, respectively, and for both loci jointly it was only 53%. In a more recent and considerably larger study of the flora of Canada, overall success rates were 35% for *matK* (*n* = 9412 specimens), 84% for *rbcL* (*n* = 20816) and 42% for ITS2 (*n* = 13233). In the latter study, a combination of two markers was successful in 51–71% of specimens, whereas all three markers were only successful in 48% of the specimens (*n* = 2442). In comparison, on average, approximately 120 genes were assembled in our study. The cost of genome skims at the start of our project was five times that of two standard barcodes. There is a breakpoint in effort and costs, where it pays off to do genome skimming rather than adding more single barcodes. Exactly where this breakpoint is depends on individual laboratories’ facilities, methods used, the complexity of the flora, the purpose of the reference library, as well as the success rate of the different approaches. However, no single barcode region can be used to identify all plant species across genera and families, and the number and type of loci needed for the individual large-scale reference library project is usually unknown at the start of a project. Thus, especially when several loci are targeted in a study, genome skimming may be more cost efficient compared to amplicon sequencing. This is particularly true if the labour costs involved in repeated attempts to sequence difficult regions such as *matK* are factored into the calculations. 

### 3.3. Success Rate Among Families

As with amplicon sequencing, we also observed some differences in success rates among families. Boraginaceae had by far the lowest success rate in our study. This family also had a low success rate for both silica gel dried and herbarium material from Canada [35]. Contrary to expectations, our herbarium material worked reasonably well in this family, with 67% success. Similarly, Nevill et al. [37] had high success for genome skims of herbarium material. Kuzmina et al. [35] suggested that low success rates in Boraginaceae could be related to the fact that most genera of this family are synthesising and storing pyrrolizidine alkaloids, compounds that may cause rapid and permanent DNA damage [51]. They further suggested that these compounds may preclude DNA preservation during the early phase of drying, immediately after collection. However, if this was the case, we would have expected better results for silica gel dried than herbarium material. The success rate of both the current study and that of Nevill et al. (2020) suggest that these compounds might inhibit DNA extraction, and that this may be broken down over time in herbarium material. 

Aspleniaceae worked well for silica gel dried but less so for herbarium material. Sanger sequencing is often also problematic for ferns [35,52]. In fact, eight and eleven fern families failed for *matK* and ITS2, respectively, in the Sanger sequencing based study of the flora of Canada [35]. In contrast, our genome skim worked for all 161 families that we tested. Also, the major problem with ferns for our data may be bioinformatic rather than sequencing success, because their sequences diverge from the Angiosperms which are mainly used in the assembling and annotating tools.

### 3.4. Effect of Age and Time of Season

We found no effect of age in up to 153-year-old herbarium material. Similarly, Nevill et al. [37] found no effect of age on genome skims of up to 80-year-old material. Also for capture probes, the target enrichment efficiency declines with age [53]. This is in contrast to what has been observed for Sanger sequencing [35,54]. Similarly, while we in an earlier Sanger sequencing study observed that specimens collected later in the growing season had poorer success rate (boldsystems.org, project NNOR, n = 1805), time of season caused no problem for genome skimming. Thus, genome skimming seems less effected by template quality than alternative methods.

### 3.5. Utilisation of the Genome Skimming Data 

Our preliminarily targeted assembly shows that most genes can be assembled with the skimming approach used here. This allows us to analyse gene dropout [44] and phylogenies [4]. Also, the taxonomic resolution of difficult taxa may be enhanced with this approach [6,13,55,56] and even within-species diversity can be studied using this protocol [31].

Our genome skims allow for the design of purpose primers and/or capture probes [57,58,59]. For example, Li et al. [20] suggest a two-step barcode process, where the plastid genome is used in a second step for designing within-group markers. As the plastid genome of 8–10 taxa may be sufficient to design taxon-specific barcodes [20], even the skimming of only a small proportion of the global flora may greatly improve the discrimination power of barcodes. Thus, our dataset of 43 families with a minimum of 20 taxa, may already greatly advance the possibilities to design primers or capture probes.

For studies of environmental DNA and ancient DNA, the genome skims allows for direct comparison with shotgun sequence data of sediments. The first 256 genome skims of the PhyloNorway dataset were used in an ancient DNA study from southern Sweden, and greatly enhanced the ability to taxonomically assign the sequences [60]. Furthermore, it may enable studies of population genomics based on herbarium material [61] or sediment samples, as has been done for algae [62] and humans [63]. 

In the near future, as the number of fully assembled genomes steadily increase, the ability to use these for assembling genome skim data will increase. This may allow for increased assembly and utilisation of the nuclear and mitochondrial DNA [64] recovered from the genome skims, increasing the power and range of applications for the data. 

## 4. Materials and Methods 

### 4.1. Sampling and DNA Extraction

For PhyloAlps, we collected most of the fresh leaves during the summer months in 2009, 2010, and 2011, with some additional materials collected in subsequent years to fill sampling gaps. Most PhyloCarpates samples were collected during the 2013, 2015 and 2016 fieldwork seasons, focusing on Carpathian endemics and regionally distributed Carpathian–Balkan taxa. 

For PhyloNorway, we sampled leaf material from herbarium specimens at Tromsø Museum (herbarium TROM, 220,000 specimens). The only treatment used for minimising insects in this herbarium is freezing at −30 °C for 4 days. The specimens in the herbarium are stored at a temperature of around 15 °C in woody cabinets with around 50% humidity. Specimens were selected using 5 criteria: (1) The species is native in boreal and/or arctic regions; (2) The specimen is healthy—every specimen was inspected under a dissecting microscope to exclude specimens with e.g., visible fungal infections.; (3) Collection date for the specimen is as early in the growing season as possible; (4) Sampling of specimens collected in the field after year 2000 was prioritised, where they met the other criteria; (5) The sample has good documentation and reliable taxonomic identification. The primary aim was to cover all species of Norway and polar regions, but common invasive plant species in this region were also included. 

DNA extractions were performed using Macherey-Nagel Nucleospin 96 Plant II kit with the following specifications and modifications. A minimum of 20 mg dried leaf material was collected from each specimen; a few specimens had less material due to their small size. Two tungsten carbide beads (3 mm diameter) were added to each sample before they were inserted into the TissueLyser for 4 × 1 minutes at 25 Hz. For each batch of 96 samples, a lysis buffer consisting of 50 mL Buffer PL1 and 1 mL RNase A were prepared, and 500 µL lysis buffer was dispensed to each sample. For silica dried samples (PhyloAlps and PhyloCarpates), a brief spin was performed at this step; this was skipped for herbarium material (PhyloNorway). Incubation time at 65 °C was increased to overnight for all samples, followed by a centrifugation step, silica gel dried material for 10 min 16,000× *g* and herbarium material for 15 min at 13,200 rpm. A filtration step was performed after step 3 in the original protocol, loading 400 μL cell lysate into NucleoSpin Flash Filter Plate stacked on top of a square-well block, and then centrifuged for 2 min at 2500× *g* for silica dried and 4600 rpm for herbarium material. Thereafter, 450 μL Binding Buffer PC was added to the square-well block. For step 6 in the original protocol (DNA binding to silica membrane), centrifugation was increased to 20 min at 4600 rpm for herbarium material. All wash steps for herbarium material were centrifuged at 4600 rpm. In step 7 (wash and dry silica membrane), all wash steps for herbarium material were centrifuged at 4600 rpm. For the third wash, we first centrifuged for 2 min before the square-well block was emptied and re-centrifuged without seal for 5 min, and then dried at room temperature for 5 min instead of the original 10 min centrifugation. For step 8 (DNA elution), we used 150 μL preheated Buffer PE and the flow-through was re-applied onto the filter to increase DNA yielding for herbarium material. See full extraction protocol in Supplementary Appendix 2.

### 4.2. Library Preparation and Sequencing 

The library preparation protocol applied was chosen on the basis of the DNA extraction yields. When available, 250 ng of genomic DNA were sonicated using the E210 Covaris instrument (Covaris, Inc., USA). The NEBNext DNA Modules Products (New England Biolabs, MA, USA) were used for end-repair, 3’-adenylation and ligation of NextFlex DNA barcodes (Bio Scientific Corporation). After two consecutive 1x AMPure XP clean ups, the ligated products were amplified by 12 cycles PCR using Kapa Hifi Hotstart NGS library Amplification kit (Kapa Biosystems, Wilmington, MA), followed by a 0.6x AMPure XP purification. When the extraction yielded low DNA quantities, 10–50 ng of genomic DNA were sonicated. Fragments were end-repaired, 3’-adenylated and NEXTflex DNA barcoded adapters were added by using NEBNext Ultra II DNA Library prep kit for Illumina (New England Biolabs). After two consecutive 1x AMPure clean ups, the ligated products were PCR-amplified with NEBNext Ultra II Q5 Master Mix included in the kit, followed by 0.8x AMPure XP purification. 

All libraries were subjected to size profile analyses conducted by Agilent 2100 Bioanalyzer (Agilent Technologies, USA) and qPCR quantification (MxPro, Agilent Technologies, USA), then sequenced using 101 base-length read chemistry in a paired-end flow cell on the Illumina HiSeq2000 sequencer (Illumina, USA). For 155 libraries, the same extract was sequencing twice either as a quality control or because the first results were poor. 

An Illumina filter was applied to remove the least reliable data from the analyses. The raw data were filtered to remove any clusters with too much intensity corresponding to bases other than the called base. Adapters and primers were removed from the whole read. Nucleotides exhibiting a low Illumina sequence quality score (below 20) were trimmed from both extremities of the read. Sequences between the second unknown nucleotide (N) and the end of the read were also removed. Reads shorter than 30 nucleotides after trimming were discarded. Finally, the reads and their mates that mapped onto run quality control sequences (PhiX genome) were removed. These trimming steps were achieved using internal software based on the FastX package [65]. 

### 4.3. Global Assembly and Annotation 

For each sample, the complete sequence of the nrDNA and of the chloroplast genome were first assembled using the Organelle Assembler [66], which is a De Bruijn graph based assembler specifically developed for the PhyloAlps and PhyloNorway projects and designed for the assembly of high copy genetic elements such as organelle genomes and nrDNA from genome skimming datasets.

The sequence data was indexed with the “oa index” using a variable length cut-off that retains 90% of the input sequences. The chloroplast protein coding genes and nrDNA from *Arabidopsis* were used to find the assembly seeds in the index sequence with the “oa seed” command. For both the chloroplast and nrDNA assemblies, the assembly graphs were constructed with “oa buildgraph” allowing up to 30 iterations for filling assembly gaps. The final assembled contigs were produced with “oa unfold” and “oa unfoldrdna” for the chloroplast and nrDNA assemblies respectively. A circular contig was attempted to be generated from the chloroplast assembly graph. However, if none could be obtained, the separate contigs were produced instead. The assembled sequences were annotated with the ORG.Annot pipeline [67]. 

### 4.4. Targeted Assembly for matK and rbcL 

As some chloroplast assemblies did not succeed for all specimens, we used the OrthoSkim pipeline [68] to retrieve the chloroplast genes for samples lacking complete assemblies (n = 1815). This pipeline consists of assembling all sequencing reads into genomic contigs and extracting all targeted genes from these contigs by mapping to close reference. For this, we formatted a database of chloroplast coding genes from our annotations by keeping all protein sequences. For each sample, assembly was performed in OrthoSkim using the SPAdes assembler [69] with the “SPAdes_assembly” mode. Afterwards, OrthoSkim selected the closest taxon for each gene of the database in the NCBI taxonomy and contigs were first mapped to this closed reference to extract matching contigs from the contigs set with a diamond [70]. Selected contigs were then mapped using exonerate [71] in order to identify the exonic regions for each gene, which were next extracted. This was implemented using the “extraction” mode with the “chloroplast_CDS” target.

### 4.5. Quality Control

For PhyloNorway, the chloroplast *rbcL* and nuclear ribosomal ITS2 barcode regions were extracted from the annotated database for quality controls. For each marker the data was uploaded to BOLD systems [72] and analyzed via the Taxon ID Tree option (visualization via a simple NJ tree). Samples that were misplaced in the tree were manually checked for misidentifications based on the uploaded herbarium material, corrected where possible or removed from the final dataset if the final identification was unclear. A total of 87 samples were corrected and 8 libraries were removed in this step. 

Additionally, the in-house quality control process that was applied to the reads that passed the Illumina quality filters included a taxonomic assignment step. For each dataset, taxonomic assignment was performed on a random sample of 20,000 reads using MegaBLAST [73], Kraken [74], or Centrifuge [75]. This allowed us to identify 35 additional PhyloNorway samples that likely corresponded to identification/sampling errors, as well as 113 PhyloAlps samples. These samples were discarded from subsequent analyses. We also tagged 42 PhyloNorway samples that were contaminated by other DNA from the environment (bacteria, fungi, birds, fish, human; contamination was 0.5–14% of total reads). These had lower success rates than the overall PhyloNorway dataset (Fisher *p* = 2.43 × 10^−4^), and we were only able to assemble the full chloroplast genome for 11 of these samples. These are kept in the overall dataset to give realistic statistics of success rate.

### 4.6. Statistical Analyses 

All success rates are calculated based on libraries. To evaluate the significance of correlations between continuous variables (coverage, insert size, age) with assembly success or preservation methods, Wilcoxon rank-sum tests were used. To estimate the correlation between success rate and preservation method, Fisher’s exact test was used. All statistical analyses were done in R version 3.6 [76].

### 4.7. Data Availability

For PhyloNorway, the full dataset of *matK*, *rbcL* and ITS2 is available on BOLD [72]. A subset of 1535 samples has been included in ongoing work (Wang et al. in prep) and the raw reads and sequence assemble will be deposited at the European Nucleotide Archive [77]. The remaining data will be released after further quality control. Metadata for the majority of specimens are provided on PhyloAlps [78].

## Figures and Tables

**Table 1:** The data analyzed for low coverage genome skims. The table lists the number of specimens sampled and analyzed overall (All) and for the two projects, PhyloAlp (including PhyloCarpates) and PhyloNorway. The three last columns list the number of specimens in the species-rich families that contain at least 20 taxa across the studied region. Number of complete genomes, average cover, and average library insert size (base pairs) is also given.

	All	PhyloAlps + Carp	Phylo Norway	All (+ 20 fam)	PhyloAlps + Carp (+ 20 fam)	PhyloNorway (+ 20 fam)
Specimens	6655	4604	2051	5893	4057	1836
Libraries	6817	4726	2091	6018	4147	1871
Families	161	158	112	43	43	43
Genera	1037	922	576	804	705	461
Taxa	5575	4437	1899	4957	3914	1689
Complete genome cpDNA	4439	3303	1136	3944	2922	1022
Average sequencing depth cpDNA	278	318	187	265	300	188
Complete nrDNA cluster	5748	4021	1727	5092	3543	1549
Average sequencing depth nrDNA	603	674	444	579	638	450
Average library insert size	316	346	249	318	350	249

## Supplementary Materials

The following are available online at https://www.mdpi.com/2223-7747/9/4/432/s1, Appendix 1: Sequencing success for all 6817 libraries of 6662 specimens studied. Appendix 2: DNA extraction protocol adapted for herbarium material.

## References

[1] Hollingsworth P.M., Forrest L.L., Spouge J.L., Hajibabaei M., Ratnasingham S., van der Bank M., Chase M.W., Cowan R.S., Erickson D.L., Plant Working Group (2009). A DNA barcode for land plants. Proc. Natl. Acad. Sci. USA.

[2] Hollingsworth P.M., Graham S.W., Little D.P. (2011). Choosing and using a plant DNA barcode. PLoS ONE.

[3] Braukmann T.W.A., Kuzmina M.L., Sills J., Zakharov E.V., Hebert P.D.N. (2017). Testing the efficacy of DNA barcodes for identifying the vascular plants of Canada. PLoS ONE.

[4] Li H.-T., Yi T.-S., Gao L.-M., Ma P.-F., Zhang T., Yang J.-B., Gitzendanner M.A., Fritsch P.W., Cai J., Luo Y. (2019). Origin of angiosperms and the puzzle of the Jurassic gap. Nat. Plants.

[5] Gitzendanner M.A., Soltis P.S., Wong G.K.-S., Ruhfel B.R., Soltis D.E. (2018). Plastid phylogenomic analysis of green plants: A billion years of evolutionary history. Am. J. Bot..

[6] Zhai W., Duan X., Zhang R., Guo C., Li L., Xu G., Shan H., Kong H., Ren Y. (2019). Chloroplast genomic data provide new and robust insights into the phylogeny and evolution of the Ranunculaceae. Mol. Phylogenet. Evol..

[7] Fahner N.A., Shokralla S., Baird D.J., Hajibabaei M. (2016). Large-scale monitoring of plants through environmental DNA metabarcoding of soil: Recovery, resolution, and annotation of four DNA markers. PLoS ONE.

[8] Liu J., Yan H.-F., Newmaster S.G., Pei N., Ragupathy S., Ge X.-J. (2015). The use of DNA barcoding as a tool for the conservation biogeography of subtropical forests in China. Divers. Distrib..

[9] Willerslev E., Davison J., Moora M., Zobel M., Coissac E., Edwards M.E., Lorenzen E.D., Vestergård M., Gussarova G., Haile J. (2014). Fifty thousand years of Arctic vegetation and megafaunal diet. Nature.

[10] Parducci L., Bennett K.D., Ficetola G.F., Alsos I.G., Suyama Y., Wood J.R., Pedersen M.W. (2017). Ancient plant DNA in lake sediments. New Phytol..

[11] Clarke C.L., Edwards M.E., Gielly L., Ehrich D., Hughes P.D.M., Morozova L.M., Haflidason H., Mangerud J., Svendsen J.I., Alsos I.G. (2019). Persistence of arctic-alpine flora during 24,000 years of environmental change in the Polar Urals. Sci. Rep..

[12] Kool A., de Boer H.J., Krüger A., Rydberg A., Abbad A., Björk L., Martin G. (2012). Molecular identification of commercialized medicinal plants in southern Morocco. PLoS ONE.

[13] Bi Y., Zhang M.-F., Xue J., Dong R., Du Y.-P., Zhang X.-H. (2018). Chloroplast genomic resources for phylogeny and DNA barcoding: A case study on *Fritillaria*. Sci. Rep..

[14] Soininen E.M., Gauthier G., Bilodeau F., Berteaux D., Gielly L., Taberlet P., Gussarova G., Bellemain E., Hassel K., Stenøien H.K. (2015). Highly overlapping winter diet in two sympatric lemming species revealed by DNA metabarcoding. PLoS ONE.

[15] Bell K.L., Burgess K.S., Okamoto K.C., Aranda R., Brosi B.J. (2016). Review and future prospects for DNA barcoding methods in forensic palynology. Forensic Sci. Int. Genet..

[16] Lang D., Tang M., Hu J., Zhou X. (2019). Genome-skimming provides accurate quantification for pollen mixtures. Mol. Ecol. Resour..

[17] Xu S.-Z., Li Z.-Y., Jin X.-H. (2018). DNA barcoding of invasive plants in China: A resource for identifying invasive plants. Mol. Ecol. Resour..

[18] Fazekas A.J., Kuzmina M.L., Newmaster S.G., Hollingsworth P.M., Kress W.J., Erickson D.L. (2012). DNA barcoding methods for land plants. DNA Barcodes: Methods and Protocols.

[19] Taberlet P., Coissac E., Pompanon F., Gielly L., Miquel C., Valentini A., Vermat T., Corthier G., Brochmann C., Willerslev E. (2007). Power and limitations of the chloroplast trnL (UAA) intron for plant DNA barcoding. Nucleic Acids Res..

[20] Li X., Yang Y., Henry R.J., Rossetto M., Wang Y., Chen S. (2015). Plant DNA barcoding: From gene to genome. Biol. Rev. Camb. Philos. Soc..

[21] Kress W.J. (2017). Plant DNA barcodes: Applications today and in the future. J. Syst. Evol..

[22] Hollingsworth P.M., Li D.-Z., van der Bank M., Twyford A.D. (2016). Telling plant species apart with DNA: From barcodes to genomes. Philos. Trans. R. Soc. Lond. B Biol. Sci..

[23] Coissac E., Hollingsworth P.M., Lavergne S., Taberlet P. (2016). From barcodes to genomes: Extending the concept of DNA barcoding. Mol. Ecol..

[24] Tonti-Filippini J., Nevill P.G., Dixon K., Small I. (2017). What can we do with 1000 plastid genomes?. Plant J..

[25] McKain M.R., Johnson M.G., Uribe-Convers S., Eaton D., Yang Y. (2018). Practical considerations for plant phylogenomics. Appl. Plant. Sci..

[26] Staats M., Erkens R.H.J., van de Vossenberg B., Wieringa J.J., Kraaijeveld K., Stielow B., Geml J., Richardson J.E., Bakker F.T. (2013). Genomic treasure troves: Complete genome sequencing of herbarium and insect museum specimens. PLoS ONE.

[27] Straub S.C.K., Fishbein M., Livshultz T., Foster Z., Parks M., Weitemier K., Cronn R.C., Liston A. (2011). Building a model: Developing genomic resources for common milkweed (*Asclepias syriaca*) with low coverage genome sequencing. BMC Genomics.

[28] Kane N.C., Cronk Q. (2008). Botany without borders: Barcoding in focus. Mol. Ecol..

[29] Parks M., Cronn R., Liston A. (2009). Increasing phylogenetic resolution at low taxonomic levels using massively parallel sequencing of chloroplast genomes. BMC Biol..

[30] Nock C.J., Waters D.L.E., Edwards M.A., Bowen S.G., Rice N., Cordeiro G.M., Henry R.J. (2011). Chloroplast genome sequences from total DNA for plant identification. Plant Biotechnol. J..

[31] Chen J.-H., Huang Y., Brachi B., Yun Q.-Z., Zhang W., Lu W., Li H.-N., Li W.-Q., Sun X.-D., Wang G.-Y. (2019). Genome-wide analysis of Cushion willow provides insights into alpine plant divergence in a biodiversity hotspot. Nat. Commun..

[32] Bendich A.J. (2004). Circular chloroplast chromosomes: The grand illusion. Plant. Cell.

[33] Kim K., Lee S.-C., Lee J., Yu Y., Yang K., Choi B.-S., Koh H.-J., Waminal N.E., Choi H.-I., Kim N.-H. (2015). Complete chloroplast and ribosomal sequences for 30 accessions elucidate evolution of Oryza AA genome species. Sci. Rep..

[34] Steele P.R., Hertweck K.L., Mayfield D., McKain M.R., Leebens-Mack J., Pires J.C. (2012). Quality and quantity of data recovered from massively parallel sequencing: Examples in Asparagales and Poaceae. Am. J. Bot..

[35] Kuzmina M.L., Braukmann T.W.A., Fazekas A.J., Graham S.W., Dewaard S.L., Rodrigues A., Bennett B.A., Dickinson T.A., Saarela J.M., Catling P.M. (2017). Using herbarium-derived DNAs to assemble a large-scale DNA barcode library for the vascular plants of Canada. Appl. Plant Sci..

[36] De Vere N., Rich T.C.G., Ford C.R., Trinder S.A., Long C., Moore C.W., Satterthwaite D., Davies H., Allainguillaume J., Ronca S. (2012). DNA barcoding the native flowering plants and conifers of Wales. PLoS ONE.

[37] Nevill P.G., Zhong X., Tonti-Filippini J., Byrne M., Hislop M., Thiele K., van Leeuwen S., Boykin L.M., Small I. (2020). Large scale genome skimming from herbarium material for accurate plant identification and phylogenomics. Plant. Methods.

[38] Pyke G.H., Ehrlich P.R. (2010). Biological collections and ecological/environmental research: A review, some observations and a look to the future. Biol. Rev. Camb. Philos. Soc..

[39] Suarez A.V., Tsutsui N.D. (2004). The value of museum collections for research and society. Bioscience.

[40] Särkinen T., Staats M., Richardson J.E., Cowan R.S., Bakker F.T. (2012). How to open the treasure chest? Optimising DNA extraction from herbarium specimens. PLoS ONE.

[41] Zeng C.-X., Hollingsworth P.M., Yang J., He Z.-S., Zhang Z.-R., Li D.-Z., Yang J.-B. (2018). Genome skimming herbarium specimens for DNA barcoding and phylogenomics. Plant Methods.

[42] Dormontt E.E., van Dijk K.-J., Bell K.L., Biffin E., Breed M.F., Byrne M., Caddy-Retalic S., Encinas-Viso F., Nevill P.G., Shapcott A. (2018). Advancing DNA barcoding and metabarcoding applications for plants requires systematic analysis of herbarium collections—An Australian perspective. Front. Ecol. Evol..

[43] Lang P.L.M., Willems F.M., Scheepens J.F., Burbano H.A., Bossdorf O. (2019). Using herbaria to study global environmental change. New Phytol..

[44] Besnard G., Christin P.-A., Malé P.-J.G., Lhuillier E., Lauzeral C., Coissac E., Vorontsova M.S. (2014). From museums to genomics: Old herbarium specimens shed light on a C3 to C4 transition. J. Exp. Bot..

[45] Zedane L., Hong-Wa C., Murienne J., Jeziorski C., Baldwin B.G., Besnard G. (2016). Museomics illuminate the history of an extinct, paleoendemic plant lineage (Hesperelaea, Oleaceae) known from an 1875 collection from Guadalupe Island, Mexico. Biol. J. Linn. Soc. Lond..

[46] Bakker F.T., Lei D., Yu J., Mohammadin S., Wei Z., van de Kerke S., Gravendeel B., Nieuwenhuis M., Staats M., Alquezar-Planas D.E. (2016). Herbarium genomics: Plastome sequence assembly from a range of herbarium specimens using an Iterative Organelle Genome Assembly pipeline. Biol. J. Linn. Soc. Lond..

[47] Elliott T.L., Jonathan Davies T. (2014). Challenges to barcoding an entire flora. Mol. Ecol. Resour..

[48] Johnson B.M., Kemp B.M. (2017). Rescue PCR: Reagent-rich PCR recipe improves amplification of degraded DNA extracts. J. Archaeol. Sci. Rep..

[49] Carøe C., Gopalakrishnan S., Vinner L., Mak S.S.T., Sinding M.H.S., Samaniego J.A., Wales N., Sicheritz-Pontén T., Gilbert M.T.P. (2018). Single-tube library preparation for degraded DNA. Methods Ecol. Evol..

[50] Gansauge M.-T., Gerber T., Glocke I., Korlevic P., Lippik L., Nagel S., Riehl L.M., Schmidt A., Meyer M. (2017). Single-stranded DNA library preparation from highly degraded DNA using T4 DNA ligase. Nucleic Acids Res..

[51] El-Shazly A., Wink M. (2014). Diversity of pyrrolizidine alkaloids in the Boraginaceae structures, distribution, and biological properties. Diversity.

[52] Sønstebø J.H., Gielly L., Brysting A.K., Elven R., Edwards M., Haile J., Willerslev E., Coissac E., Rioux D., Sannier J. (2010). Using next-generation sequencing for molecular reconstruction of past Arctic vegetation and climate. Mol. Ecol. Resour..

[53] Brewer G.E., Clarkson J.J., Maurin O., Zuntini A.R., Barber V., Bellot S., Biggs N., Cowan R.S., Davies N.M.J., Dodsworth S. (2019). Factors affecting targeted sequencing of 353 nuclear genes from herbarium specimens spanning the diversity of Angiosperms. Front. Plant. Sci..

[54] Korpelainen H., Pietiläinen M. (2019). The effects of sample age and taxonomic origin on the success rate of DNA barcoding when using herbarium material. Plant. Syst. Evol..

[55] Malé P.-J.G., Bardon L., Besnard G., Coissac E., Delsuc F., Engel J., Lhuillier E., Scotti-Saintagne C., Tinaut A., Chave J. (2014). Genome skimming by shotgun sequencing helps resolve the phylogeny of a pantropical tree family. Mol. Ecol. Resour..

[56] Gryta H., Van de Paer C., Manzi S., Holota H., Roy M., Besnard G. (2017). Genome skimming and plastid microsatellite profiling of alder trees (*Alnus* spp., Betulaceae): Phylogenetic and phylogeographical prospects. Tree Genet. Genomes.

[57] De La Harpe M., Hess J., Loiseau O., Salamin N., Lexer C., Paris M. (2019). A dedicated target capture approach reveals variable genetic markers across micro- and macro-evolutionary time scales in palms. Mol. Ecol. Resour..

[58] Schmid S., Genevest R., Gobet E., Suchan T., Sperisen C., Tinner W., Alvarez N. (2017). HyRAD-X, a versatile method combining exome capture and RAD sequencing to extract genomic information from ancient DNA. Methods Ecol. Evol..

[59] Schulte L., Bernhardt N., Stoof-Leichsenring K.R., Zimmermann H.H., Pestryakova L.A., Epp L.S., Herzschuh U. (2020). Hybridization capture of larch (*Larix* Mill) chloroplast genomes from sedimentary ancient DNA reveals past changes of Siberian forests. bioRxiv.

[60] Parducci L., Alsos I.G., Unneberg P., Pedersen M.W., Han L., Lammers Y., Salonen J.S., Valiranta M.M., Slotte T., Wohlfarth B. (2019). Shotgun environmental DNA, pollen, and macrofossil analysis of Lateglacial lake sediments from southern Sweden. Front. Ecol. Evol..

[61] Johnson J.S., Krutovsky K.V., Rajora O.P., Gaddis K.D., Cairns D.M., Rajora O.P. (2019). Advancing biogeography through population genomics. Population Genomics: Concepts, Approaches and Applications.

[62] Lammers Y. (2020). Sedimentary ancient DNA: Exploring Methods of Ancient DNA Analysis for Different Taxonomic Groups. Ph.D. Thesis.

[63] Slon V., Hopfe C., Weiß C.L., Mafessoni F., de la Rasilla M., Lalueza-Fox C., Rosas A., Soressi M., Knul M.V., Miller R. (2017). Neandertal and Denisovan DNA from Pleistocene sediments. Science.

[64] Berger B.A., Han J., Sessa E.B., Gardner A.G., Shepherd K.A., Ricigliano V.A., Jabaily R.S., Howarth D.G. (2017). The unexpected depths of genome-skimming data: A case study examining Goodeniaceae floral symmetry genes. Appl. Plant. Sci..

[65] FastX Package. http://hannonlab.cshl.edu/fastx_toolkit/index.html.

[66] Organelle Assembler. http://metabarcoding.org/asm.

[67] ORG.Annot Pipeline. https://metabarcoding.org/annot.

[68] OrthoSkim Pipeline. https://github.com/cpouchon/OrthoSkim.

[69] Bankevich A., Nurk S., Antipov D., Gurevich A.A., Dvorkin M., Kulikov A.S., Lesin V.M., Nikolenko S.I., Pham S., Prjibelski A.D. (2012). SPAdes: A new genome assembly algorithm and its applications to single-cell sequencing. J. Comput. Biol..

[70] Buchfink B., Xie C., Huson D.H. (2015). Fast and sensitive protein alignment using DIAMOND. Nat. Methods.

[71] Slater G.S.C., Birney E. (2005). Automated generation of heuristics for biological sequence comparison. BMC Bioinform..

[72] BOLD Systems. https://www.boldsystems.org.

[73] Zhang Z., Schwartz S., Wagner L., Miller W. (2000). A greedy algorithm for aligning DNA sequences. J. Comput. Biol..

[74] Wood D.E., Salzberg S.L. (2014). Kraken: Ultrafast metagenomic sequence classification using exact alignments. Genome Biol..

[75] Kim D., Song L., Breitwieser F.P., Salzberg S.L. (2016). Centrifuge: Rapid and sensitive classification of metagenomic sequences. Genome Res..

[76] R_Core_Team (2013). R: A Language and Environment for Statistical Computing.

[77] European Nucleotide Archive (ENA-EBI). https://www.ebi.ac.uk/ena.

[78] PhyloAlps. https://data.phyloalps.org/browse/.

