# Supplementary material for: The Treasure Vault Can be Opened: Large-Scale Genome Skimming Works Well Using Herbarium and Silica Gel Dried Material
Source: Plants (Basel). 2020 Apr 1;9(4):432. doi: 10.3390/plants9040432 (PMC7238428; doi:10.3390/plants9040432)
Supplement: Supplementary file 1 [file plants-09-00432-s001.zip › plants-744983-supplementary-for conversion/Appendix 2.docx]

Appendix 2

Extraction protocol for herbarium material according to MACHEREY-NAGEL – 05 / 2014, Rev. 03 and our modifications

NucleoSpin 96 Plant II – centrifuge processing

Before Starting Step 1

Check if Buffer PW2 and RNase A are prepared:

Wash Buffer PW2: Add the indicated volume of ethanol (96–100%) to Buffer PW2 concentrate before first use. Buffer PW2 may be stored at RT (18–25 °C) for up to one year.

RNase A: Add the given volume of water (indicated on the vial) to lyophilised RNase A. The RNase A solution may be stored at 4 °C for up to 3 months. For longer storage (up to 1 year), the RNase A solution should be divided into small aliquots and stored at −20 °C.

1. Homogenise Sample

Homogenise minimum 20 mg dry weight plant material. Do not exceed this weight much.

Place the tissues in 2 mL microcentrifuge tubes containing 2 tungsten carbide bead (3 mm mean diameter).

Operate the TissueLyser for 1 min at 25 Hz. Disassemble the adapter set, rotate the rack of tubes so that the tubes nearest to the TissueLyser are now outermost, and reassemble the adapter set. Operate the TissueLyser for another 1 min at 25 Hz. Repeat this step (4 × 1 min).

2. Cell Lysis

Add 575 μL Buffer PL1 and 11,5 μL RNase A. For 2 × 96 samples, mix 100 mL Buffer PL1 and 2 mL RNase A, and dispense 500 μL mix to each sample. Some samples needs more buffer due to liquid is taken up into the cells.

Vortex the mixture thoroughly.

Shake up and down to collect any sample from the caps.

Incubate the suspension overnight at 65 °C (oven). Turn the samples upside down at least once before going home for the day to make sure you get what is laying in the lid.

Before Starting Step 3

Equilibrate Buffer PE to 70 °C (around 40 mL will be used for two plates)

3. Clear Lysate by Centrifugation and Filtration

Centrifuge the samples for 15 min at 13 000 rpm

Place the NucleoSpin Flash Filter Plate on a fresh Square-well Block.

Load 400 μL cell lysate into the wells of the NucleoSpin Flash Filter Plate.

Seal openings of the filter plate with a Gas-permeable Foil.

Centrifuge for 2 min at 4600 rpm

4. Adjust Binding Conditions

Place the NucleoSpin Plant II Binding Plate stacked on the washing Square-well Block.

Add 450 μL Binding Buffer PC to each samples in the well of the Square-well Block, mix by repeated pipetting up and down.

5. Transfer Lysate to NucleoSpin® Plant II Binding Plate

Place a new NucleoSpin Plant II Binding Plate on a new Square-wll Block and transfer the samples (<900 μL) into the wells of the NucleoSpin Plant II Binding Plate. Do not moisten the rims of the individual wells while dispensing the samples.

Seal openings of the binding plate with a Gas-permeable Foil.

6. Bind DNA to Silica Membrane

Centrifuge at 4600 rpm for 20 min. Typically, lysates will pass through the columns within 1 min. The process can be extended to 20 min, if the lysates have not passed completely. Empty block

Tips: PC buffer stays better in the pipette tips if the pipette is wetted by PC buffer before transferring the liquid.

7. Wash Silica Membrane

*1^st^ wash*

Add 400 μL PW1 to each well of the NucleoSpin Plant II Binding Plate.

Seal plate with a Gas-permeable Foil.

Centrifuge at 4600 rpm for 2 min. Empty the Square-well Block.

*2^nd^ wash*

Add 700 μL PW2 to each well of the NucleoSpin Plant II Binding Plate.

Seal plate with a Gas-permeable Foil.

Centrifuge at 4600 rpm for 2 min empty block

*3^rd^ wash*

Add 700 μL PW2 to each well of the NucleoSpin Plant II Binding Plate.

Seal plate with a Gas-permeable Foil.

Centrifuge at 4600 rpm for 2 min. Empty the Square-well Block.

Remove the Gas-permeable foil and centrifuge again at 4600 rpm for 5 min.

Let the NucleoSpin Plant II Binding Plate dry 5 -10 min at room temperature.

8. Elute DNA

Place NucleoSpin Plant II Binding Plate on clean square well block

Dispense 150 μL pre-heated Buffer PE (70 °C) to each well of the NucleoSpin Plant II Binding Plate. Dispense the buffer directly onto the membrane.

Incubate for 2 min at 70 °C before centrifugation.

Centrifuge at 4600 x g for 2 min.

Transfer the liquid containing the DNA one more time to the filter and centrifuge for 2 min. This is to increase the yield and concentration. Incubation is not needed.

Sometimes all liquid does not go through every filter. Process as mentioned above but after the second run, place the filters on a suitable block, add 50 uL elution buffer, and centrifuge for 10–20 min. This is to avoid messing with the samples that worked and you get an additional backup with very, very low concentration of the other samples that worked.
